# Alpha/Beta-Hydrolase Domain-Containing 6: Signaling and Function in the Central Nervous System

**DOI:** 10.3389/fphar.2021.784202

**Published:** 2021-12-02

**Authors:** Haofuzi Zhang, Xin Li, Dan Liao, Peng Luo, Xiaofan Jiang

**Affiliations:** ^1^ Department of Neurosurgery, Xijing Hospital, Fourth Military Medical University, Xi’an, China; ^2^ Department of Anesthesiology, Xijing Hospital, Fourth Military Medical University, Xi’an, China

**Keywords:** ABHD6, endocannabinoid system, CPT1C, AMPA receptor, neurological diseases

## Abstract

Endocannabinoid (eCB) signaling plays an important role in the central nervous system (CNS). α/β-Hydrolase domain-containing 6 (ABHD6) is a transmembrane serine hydrolase that hydrolyzes monoacylglycerol (MAG) lipids such as endocannabinoid 2-arachidonoyl glycerol (2-AG). ABHD6 participates in neurotransmission, inflammation, brain energy metabolism, tumorigenesis and other biological processes and is a potential therapeutic target for various neurological diseases, such as traumatic brain injury (TBI), multiple sclerosis (MS), epilepsy, mental illness, and pain. This review summarizes the molecular mechanisms of action and biological functions of ABHD6, particularly its mechanism of action in the pathogenesis of neurological diseases, and provides a theoretical basis for new pharmacological interventions *via* targeting of ABHD6.

## Introduction

The endocannabinoid system (ECS) is a lipid signal transduction system that includes endogenous cannabinoids (eCBs), cannabinoid receptors and enzymes responsible for the synthesis and hydrolyzation of eCBs, with an important role in the regulation of central nervous system (CNS) function (Jung et al., 2021; Lu and Mackie, 2021). α/β-Hydrolase domain-containing 6 (ABHD6) is an integral membrane protein with recently discovered serine hydrolase activity that is, mainly expressed in immune cell-enriched tissues and the CNS(Poursharifi et al., 2017). The hydrolytic substrates of ABHD6 are mainly monoacylglycerols (MAGs) (Poursharifi et al., 2020), such as endogenous cannabinoid 2-arachidonoylglycerol (2-AG) and arachidonic acid (Noguchi et al., 2021), which are precursors of prostaglandins and other inflammatory mediators (Ghosh et al., 2020; Larsson et al., 2021; Trostchansky et al., 2021). As the main hydrolase of postsynaptic eCBs, ABHD6 is involved in regulating neuronal ECS function (Cao et al., 2019). In addition, ABHD6 is an important component of the α-amino-3-hydroxy-5-methyl-4-isoxazole-propionic acid receptor (AMPAR) complex (Schwenk et al., 2019), which inhibits translocation of AMPAR to the postsynaptic membrane and the excitability of AMPAR (Wei et al., 2017). ABHD6 plays a potential regulatory role in the neuroinflammatory pathway (Tanaka et al., 2017; Wen et al., 2018) and autoimmunity (Manterola et al., 2018b; Rahaman et al., 2019) and is expected to become a new target for intervention in nervous system diseases (Kind and Kursula, 2019). Inhibitors of ABHD6 have been explored within analgesia, anti-anxiety, anti-inflammatory contexts, as well as other areas, and are therefore promising in the treatment of inflammatory and degenerative diseases of the CNS (Deng and Li, 2020b). In this review, the latest research progress on ABHD6 for the treatment of nervous system diseases is reviewed in terms of its molecular structure, expression, and mechanism of action in the CNS.

## The Endocannabinoid System and Identification of ABHD6

### The Endocannabinoid System

The canonical endogenous cannabinoid system consists of cannabinoid receptors, signaling lipids called eCBs, and enzymes that produce and inactivate eCBs. Some atypical receptors, including several transient receptor potential (TRP) channels, G protein-coupled receptor 55 (GPR55), and glycine receptors, have also been discovered. The concept of this signaling system originated from study of the biological activity of Δ-9-tetrahydrocannabinol (THC) (Cao et al., 2019). Three decades ago, researchers discovered the ECS when seeking to identify a cannabinoid receptor that interacts with the psychoactive compounds in cannabis. Since then, research on eCBs has exploded, and more receptors, their lipid mediators and signaling pathways have been revealed (Kilaru and Chapman, 2020). Evidence-based studies have shown that eCBs regulate various aspects of human physiological and pathophysiological functions in the CNS and immune system, which has become a hot research topic (Joshi and Onaivi, 2019). The biological effects of eCBs are mediated mainly by two members of the G protein-coupled receptor family: CB_1_R and CB_2_R. CB_1_R is widely considered a potential therapeutic target in neuropsychological and neurodegenerative diseases; CB_2_R selectively regulates the function of immune cells. In addition, cannabinoids regulate signal transduction pathways and have profound roles in peripheral regions. Although cannabinoids possess therapeutic potential, their psychoactive effect limits their clinical application to a great extent (Zou and Kumar, 2018). In particular, activation of CB_1_R may cause CNS effects. Nevertheless, the discovery of CB_2_R and endogenous cannabinoid receptor ligands (namely, eCBs) provides new possibilities for safely targeting the ECS (Cristino et al., 2020). Notably, 2-AG is one of the ligands that can be metabolized by several hydrolytic enzymes. In this review, we focus particular attention to the hydrolase ABHD6.

### Identification of ABHD6

A vital component of the ECS, ABHD6 is an enzyme that regulates hydrolysis of 2-AG, which is an endogenous signaling lipid that activates CB_1_R and CB_2_R and is an important lipid precursor of the eicosanoic acid signaling pathway (Sugiura et al., 1995). 2-AG is generally believed to be metabolized to arachidonic acid (AA) and glycerol by monoacylglycerol lipase (MAGL); recent evidence suggests that other lipases, such as ABHD6 and ABHD12, are also involved in 2-AG degradation in many tissues. Among these lipases, MAGL is responsible for approximately 85% of the 2-AG degradation occurring in the CNS, whereas ABHD6 and ABHD12 account for approximately 4 and 9% of 2-AG hydrolysis, respectively (Savinainen et al., 2012). As MAGL, ABHD6, and ABHD12 are distributed is different manners and have different subcellular localizations, they regulate 2-AG hydrolysis at different times and sites. ABHD6 is an important cell signaling regulator not only in the CNS but also in peripheral tissues, with an important role in the pathogenesis of many diseases, such as metabolic syndrome (Thomas et al., 2013), obesity (Zhao et al., 2016), and autoimmune diseases and cancer (Yu et al., 2016). Genetic and pharmacological studies have revealed the therapeutic potential of ABHD6, making it an attractive target for the treatment of various diseases (Naydenov et al., 2014).

## Biochemical Characterization and Expression Profile of ABHD6

### Biochemical Characterization of ABHD6

**FIGURE 1:**
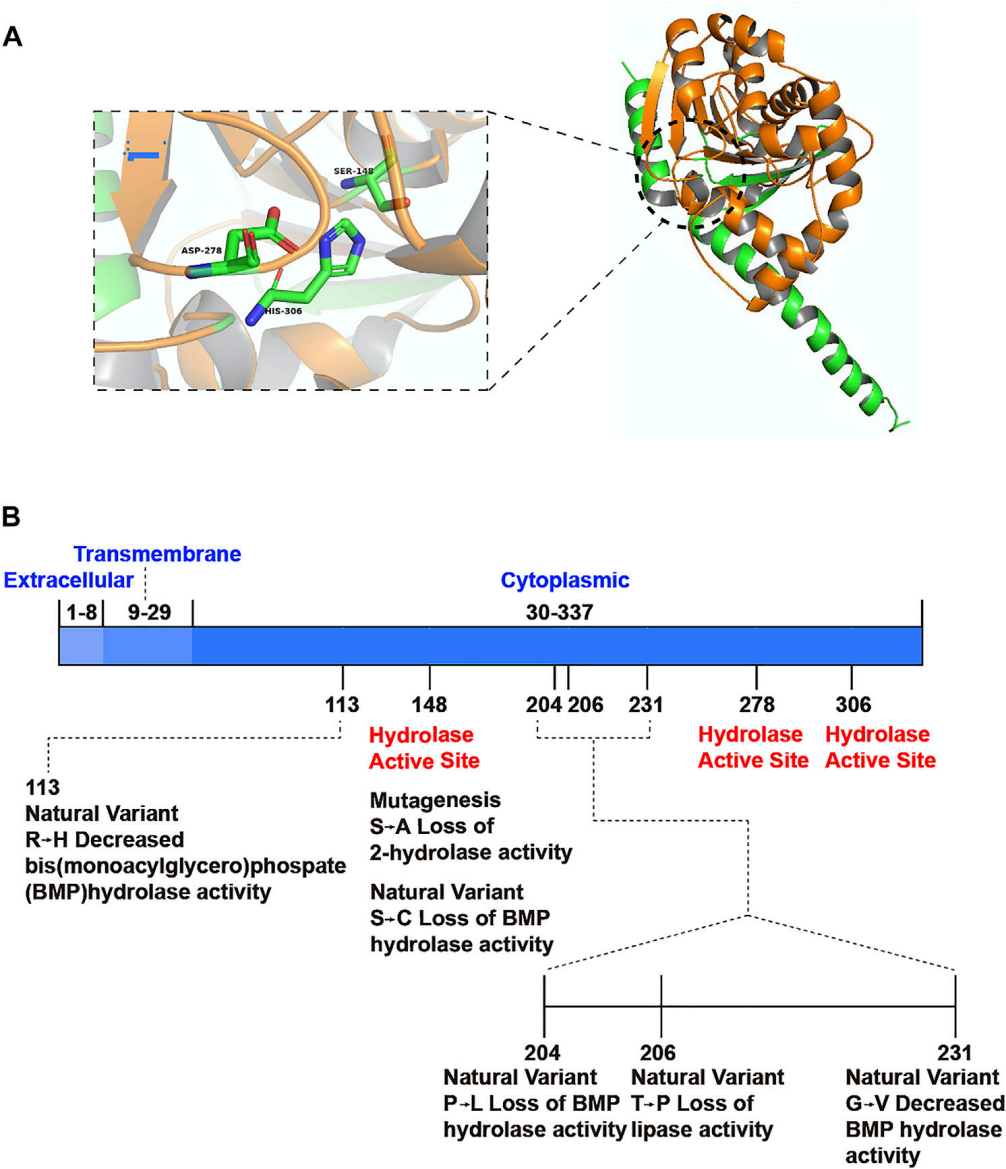
Spatial structure model of ABHD6 and its functional sites. **(A)** ABHD6 consists of an eight chain β-sheet with surrounding α-helices. The catalytic triad is composed of a nucleophilic residue (Ser148), an acidic residue (Asp278) and a histidine (His306). **(B)** Mutation or natural variation may occur at some sites of ABHD6, resulting in the decrease or loss of partial hydrolytic function of the enzyme. There is no document report that ABHD6 has modification sites on protein structures.

The ABHD6 gene is located on chromosome 3 p14.3 and consists of 10 exons. Its open reading frame encodes a protein with 337 amino acids and a molecular weight of 38 kDa (Poursharifi et al., 2017). Based on amino acid sequence, ABHD6 is a cytoplasmic type II integrated membrane protein belonging to the serine hydrolase family (Long and Cravatt, 2011). ABHD6 includes an eight-stranded parallel α/β-structure with a second antiparallel structure. The hydrolytic activity of ABHD6 derives from the catalytic triad Ser148-Asp278-His306 (Lord et al., 2013) located on the cytoplasmic side of the cell membrane (Figure 1) (Kind and Kursula, 2019). To confirm the accuracy of the predicted structure, researchers have assessed the activity of the enzyme and invoked a multidimensional protein identification platform known as activity-based protein profiling (ABPP). Site-directed mutation of the catalytic residues leads to loss of hydrolytic activity (Figure 1) (Thomas et al., 2013). Although the crystal structure of ABHD6 remains unclear, the primary structure is well conserved among species, with 94% sequence homology between human and mouse orthologs (Long and Cravatt, 2011).

The order of hydrolytic activity of ABHD6 is 1-arachidonoylglycerol (1-AG) > 2-AG > 2-LG, as measured *in vitro* using homogenate from an overexpression system (Navia-Paldanius et al., 2012). 1-AG is a similarly bioactive isomer of 2-AG that may have a role in stabilizing the strength of the cannabinoid signal (Docs et al., 2017). In primary neuronal cell culture, chemical inhibition of ABHD6 reduces 2-AG degradation by 50% and leads to 2-AG accumulation. In addition to its established MAG lipase activity, ABDH6 exhibits significant diacylglycerol lipase (DGL) activity in Neuro2A cells (van Esbroeck et al., 2019). DGLs mainly catalyze “on-demand” biosynthesis of bioactive MAGs, including 2-AG,2-linoleoylglycerol (2-LG) (Yuan et al., 2016). Because hydrolysis of 2-AG leads to release of AA, the precursor of prostaglandins, ABHD6 may be related to the inflammatory process and autoimmune diseases. Indeed, blocking ABHD6 in macrophages has anti-inflammatory effects, such as increasing the anti-inflammatory agent prostaglandin-d2-glycerol ester (PGD2-GE) (Alhouayek et al., 2013). Furthermore, ABHD6 regulates glucose-stimulated insulin secretion (GSIS), which is essential for glucose homeostasis in different tissues. A variety of different ABHD6 inhibitors have been developed through clinical disease treatment research. However, mutation of the gene encoding ABHD6 is not related to human diseases (Lord et al., 2013).

### Expression Profile of ABHD6

ABHD6 is widely expressed, especially in the CNS; enzymatic activity measured using ABPP in the mouse brain showed particular expression in the cerebral cortex (Moreno-Luna et al., 2021), pituitary (Cao et al., 2019), and hippocampus (Longaretti et al., 2020a), as well as in the spleen and small intestine and the liver, kidney, and ovary (Drehmer et al., 2019; Deng and Li, 2020b). In the CNS, ABHD6 has been identified as the key regulatory point of the ECS (Gokce et al., 2016; Cao et al., 2019). Expression of ABHD6 is regulated by estrogen and other hormones, suggesting sex differences (Drehmer et al., 2019). The distribution of ABHD6 differs in various brain regions and neural cell subtypes. Studies have shown the highest enzyme activity of ABHD6 in the frontal cortex, hippocampus, striatum, and cerebellum (Baggelaar et al., 2017). ABHD6 activity in these regions is much higher than that of ABHD12 and even that of MAGL. Although MAGL is the main enzyme related to 2-AG degradation in the brain, the role of ABHD6 is independent of MAGL and controls accumulation of 2-AG in intact neurons (Marrs et al., 2010; Owens et al., 2017). Moreover, results of single-cell sequencing showed that ABHD6 is mainly distributed in progenitor cells and astrocytes in young mice; in adult mice, it is mainly expressed in specific types of neurons (such as GABAergic neurons) and astrocytes (Marrs et al., 2010; Gokce et al., 2016). At the subcellular level, ABHD6 is mainly expressed in the cytoplasm of glutamatergic neuron dendrites and colocalizes with microtubule-associated protein 2 (MAP2), suggesting that ABHD6 plays an important role in the regulation of postsynaptic nerve function (Marrs et al., 2010). In addition, ABHD6 is significantly expressed in dendrites and postsynapses, which complements the presynaptic expression of MAGL (Straiker and Mackie, 2007), and the observed localization of ABHD6 and MAGL suggests that each enzyme controls a subcellular pool of 2-AG.

## Molecular Mechanism of ABHD6-Specific Signaling

### ABHD6 and ECS

**FIGURE 2:**
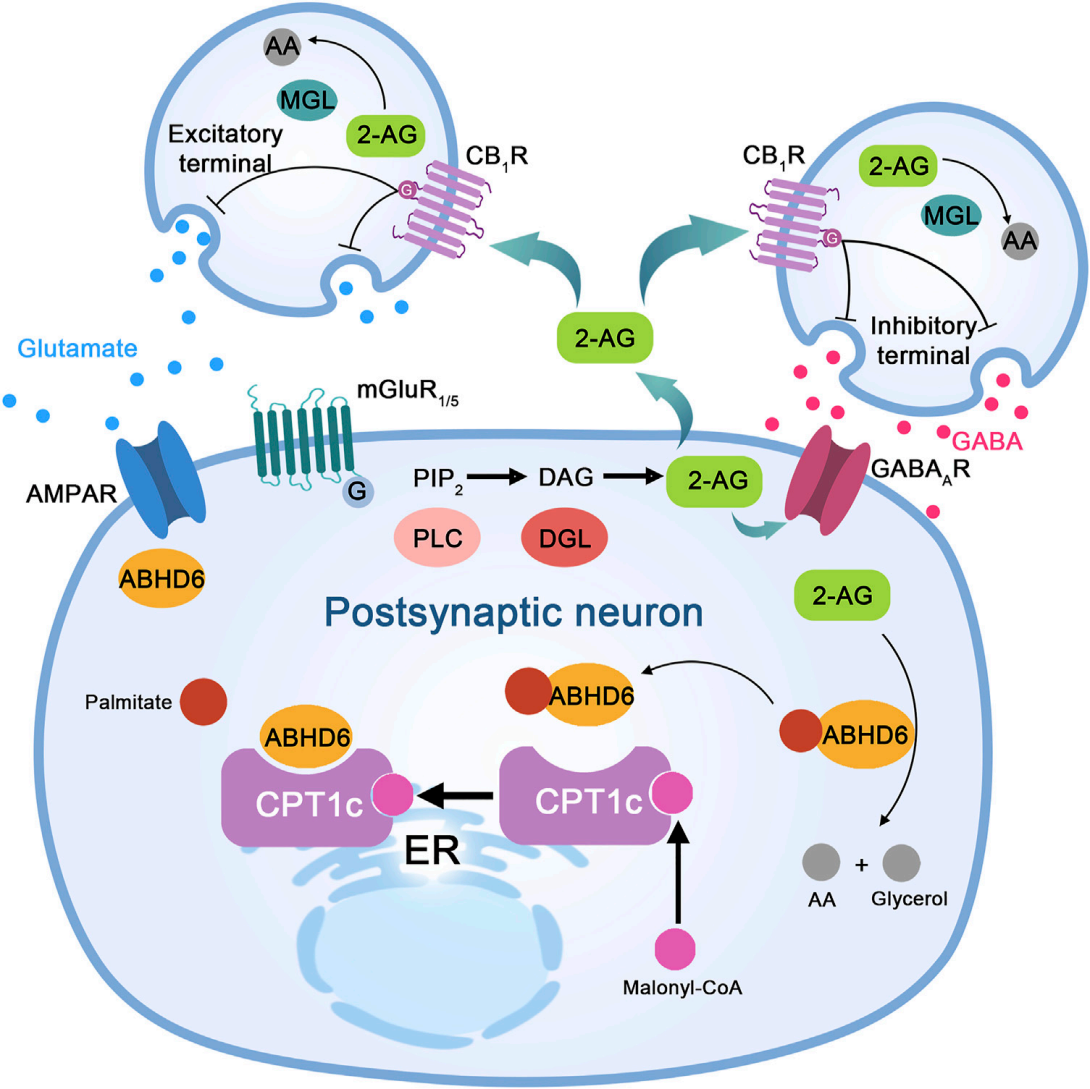
The proposed molecular mechanism of ABHD6 in neurons and neurological diseases. Stimulation of excitatory neurons to release glutamate activates metabotropic glutamate receptor 1/5 (mGluR1/5) on postsynaptic neurons, which couples to a G protein, activates phospholipase C (PLC), and leads to diacylglycerol (DAG) production from inositol diphosphate (PIP2). DAG is cleaved by DAG lipase (DGL) to produce 2-arachidonic acid glycerol (2-AG). Its functions are as follows: (I) acting as a paracrine agonist of the CB1 receptor (CB_1_R) expressed at the excitatory end, releasing glutamate to activate mGluR1/5 and α-amino-3-hydroxy-5-methyl-4-isoxazolpropionic acid (AMPA) receptors; (II) CB_1_R paracrine agonists release GABA and activate GABAA receptor (GABAAR) on postsynaptic neurons through inhibitory terminal expression; and (III) autocrine positive allosteric regulators of GABAA receptors in postsynaptic neurons. Monoacylglycerol lipase (MGL) in presynaptic neurons or α/β-hydrolase domain-containing 6 (ABHD6) in postsynaptic neurons hydrolyzes excess 2-AG to arachidonic acid (AA) and glycerol (not shown). ABHD6 interacts with the AMPA receptor and controls its transport to the postsynaptic compartment membrane. Bioinformatic analysis indicates the presence of amino acid sites of palmitoylation/depalmitoylation modification in ABHD6. Protein interaction between CPT1c and ABHD6 depends on the presence of malonyl-COA; when malonyl-COA is absent, the inhibitory effect of CPT1c on ABHD6 enzyme activity is lost.

As an important hydrolase of 2-AG, ABHD6 is directly involved in regulating the ECS. In turn, ECS regulation by ABHD6 can affect the synaptic plasticity of neurons and play a role in CNS-injuring diseases such as epilepsy and brain injury (Deng and Li, 2020b). Muccioli et al. found that BV-2 cells do not express MAGL but can effectively hydrolyze 2-AG (Muccioli et al., 2007), proving for the first time that an enzyme other than MAGL can hydrolyze 2-AG. Further studies revealed that ABHD6 acts as a 2-AG hydrolase in BV-2 cells and is a novel type of 2-AG hydrolase (Marrs et al., 2010). In addition to its expression in the BV-2 cell line, ABHD6 is expressed in neurons and astrocytes (Figure 2). Inhibition of its expression or activity enhances activation of CB_1_R and CB_2_R upon 2-AG accumulation (Marrs et al., 2010). Marrs, W.R. and others first proposed that ABHD6 participates in long-term synaptic depression (Ltd.). Their study revealed that WWL-70, an ABHD6 inhibitor, reduces the Ltd. threshold of glutamate synapses, lasting for at least 40 min after induction (Marrs et al., 2010; Marrs et al., 2011). In contrast, WWL-70 has no effect on short-term synaptic plasticity, such as depolarization-induced suppression of inhibition (DSI) or depolarization-induced suppression of excitation (DSE) (Straiker et al., 2009; Colmers and Bains, 2018). *In vitro* recordings of neurons have shown that MAGL and cyclooxygenase-2 (COX-2), but not ABHD6, mediate this process (Straiker et al., 2009; Colmers and Bains, 2018; Lopez and Ballaz, 2020). These studies distinguish the biological roles of ABHD6, MAGL, and COX-2 by emphasizing their involvement in short-term and long-term synaptic plasticity.

### ABHD6 and AMPAR

AMPAR is the major postsynaptic glutamate receptor that mediates synaptic transmission. ABHD6 specifically reduces surface expression of postsynaptic AMPARs through a hydrolase-independent mechanism (Figure 2). By using high-resolution proteomics, one study revealed that ABHD6 is a component of the AMPAR macromolecular complex (Wei et al., 2016; Chen and Gouaux, 2019). Decreased surface expression of AMPAR is due to a reduction in surface expression of GluA1, a subunit of AMPAR. Binding of ABHD6 to AMPAR subunits (GluA1, GluA2, and GluA3) is mediated by the C-terminus of subunit GluA1 (Wei et al., 2017). However evidence shows that mutation of the Ser148 site, which is crucial for the serine hydrolytic activity of ABHD6, does not affect the ability of ABHD6 to regulate postsynaptic membrane expression of AMPAR in either neurons or transfected HEK293T cells, indicating that the ABHD6-AMPAR association is endocannabinoid independent (Wei et al., 2016). ABHD6 downregulates glutamate signaling by regulating functional expression of AMPAR, which differs from its eCB-dependent effect of enhancing sensitivity to Ltd. (Wei et al., 2016). Therefore, the effects of ABHD6 on the long-term plasticity of excitatory synapses might be mediated by two distinct mechanisms: an eCB-dependent and an eCB-independent mechanism (Cao et al., 2019).

### ABHD6 and CPT1C

Carnitine palmitoyl transferase 1c (CPT1c) is a member of the carnitine palmitoyl transferase 1 family (which includes CPT1a and CPT1b), participating in the regulation of physiological functions such as energy metabolism and feeding (Obici et al., 2003). Proteomic studies have revealed that CPT1c and ABHD6 may interact (Brechet et al., 2017; Schwenk et al., 2019), and Miralpeix et al. confirmed this through coimmunoprecipitation (co-IP) and fluorescence resonance energy transfer (FRET) analyses (Miralpeix et al., 2021). Furthermore, the content of eCB in CPT1c-knockout (KO) mice is decreased (Lee and Wolfgang, 2012), indicating that interaction between CPT1c and ABHD6 may be involved in eCB hydrolysis. Further studies have confirmed that CPT1c has a significant effect on the enzymatic activity of ABHD6. Upon CPT1c overexpression, ABHD6 enzyme activity is decreased by approximately 60%, whereas activity increases significantly in CPT1c-KO mice (Miralpeix et al., 2021). Protein interaction between CPT1c and ABHD6 depends on malonyl-CoA (Figure 2). When the malonyl-CoA level is deficient, inhibition of ABHD6 enzyme activity by CPT1c disappears, indicating that cell energy metabolism can affect ABHD6 enzyme activity through the malonyl-CoA/CPT1c axis (Miralpeix et al., 2021). Overall, expression of CPT1c in tumor cells increases significantly. Moreover, the tolerance of tumor cells with high expression of CPT1c to ischemia and hypoxia is significantly enhanced (Zaugg et al., 2011; Reilly and Mak, 2012), and inhibiting expression of CPT1c has the opposite effect (Sanchez-Macedo et al., 2013). High CPT1c expression is related to enhanced cell viability under energy-deficient conditions.

## Biological Function of ABHD6

### ABHD6 and Neurotransmission

As a bona fide member of the ECS, ABHD6 regulates neuronal function through different mechanisms. DSI and DSE are mainly regulated by 2-AG degradation. Therefore, blocking hydrolysis of 2-AG may help to prolong the time course of DSI and DSE (Chevaleyre et al., 2006). Regardless, it is noteworthy that inactivation of MAGL induces DSE prolongation at Purkinje cell synapses in granule cells and olivary nuclei (Zhong et al., 2011) but that inhibition of ABHD6 has no effect on DSI or DSE in autapse preparations (Straiker et al., 2009; Straiker and Mackie, 2009). In addition, overexpression of ABHD6 or ABHD12 does not affect DSEs (Straiker and Mackie, 2009). Conversely, pharmacological inhibition of ABHD6 reduces the threshold of CB1-dependent Ltd. at glutamatergic synapses in murine cortical slices (Marrs et al., 2011). ABHD6 negatively regulates the surface transmission and synaptic function of AMPAR in neurons (Wei et al., 2016), and the physiological function of ABHD6 binding to AMPAR is considered to be independent of eCBs. These findings provide a deeper understanding of the molecular mechanism by which ABHD6 regulates synaptic AMPAR trafficking.

### ABHD6 and Neuroinflammation

Neuroinflammation is related to many neurological diseases, such as epilepsy and neurodegenerative diseases (including multiple sclerosis, Alzheimer’s disease and Parkinson’s disease), and is also linked to traumatic brain injury and ischemia-induced nerve injury. The ECS is thought to be associated with the inflammatory process and the immune system, as CB_2_R overexpression has been detected in immune cells (Hubler and Kennedy, 2016). In fact, in mouse models of experimental autoimmune encephalomyelitis and traumatic brain injury, ABHD6 inhibition reduces microglial reactivity and COX-2 expression (Martinez-Torres et al., 2019). Pharmacological blockade of ABHD6 raises the level of 2-AG, especially in microglia and macrophages (Wen et al., 2018); 2-AG inhibits infiltration of immune cells into the CNS, resulting in long-term beneficial effects in the chronic phase of autoimmune encephalomyelitis (Mecha et al., 2018). Arachidonic acid (AA), the product of 2-AG hydrolysis, is the main precursor in proinflammatory prostaglandin synthesis (Ricciotti and FitzGerald, 2011). Compared with inhibition of MAGL, also a potential target for inflammatory disease (Deng and Li, 2020a), ABHD6 inhibition has fewer side effects (detailed in subsection 5, *ABHD6 and Neurological Diseases*). Therefore, ABHD6 regulates hydrolysis of 2-AG, rendering ABHD6 a promising anti-inflammatory therapeutic target in the CNS.

### ABHD6 and Energy Homeostasis

Energy homeostasis is achieved through complex brain circuits that strictly maintain energy levels by affecting food intake and energy consumption. In general, interactions between the brain and different adipose depots play a key role in maintaining energy balance, facilitating survival, and coping with metabolic challenges such as cold exposure and starvation. The brain regulates the metabolism of brown adipose tissue (BAT), white adipose tissue (WAT), and beige adipose tissue (BeAT) through efferent pathways, and in turn, these tissues transmit information about energy storage status to the brain through sensory innervation and hormone secretion (Caron et al., 2018). It is well known that eCBs are endogenous agonists of cannabinoid receptors that regulate various physiological processes, including metabolism and food intake (Nicholson et al., 2015). The ECS is expected to regulate feed and energy consumption through ventromedial hypothalamic neurons (Busquets-Garcia et al., 2015). For example, Fisette et al. reported that mice lacking ABHD6 in neurons of the ventromedial hypothalamus (VMH) with higher VMH 2-AG levels under conditions of eCB recruitment were physiologically unable to adapt to critical metabolic challenges, suggesting that ABHD6 in the VMH is very important for flexible regulation of energy metabolism (Fisette et al., 2016). Additionally, some studies have shown that ABHD6 is a negative regulator of WAT thermogenesis (Poursharifi et al., 2020). These studies implicate the important role of ABHD6 in the regulation of energy homeostasis by modulating brain circuits.

### ABHD6 and Tumors

It has been reported that expression of ABHD6 is related to the pathogenesis of Epstein-Barr virus (EBV)-associated malignant tumors, such as Hodgkin’s lymphoma, endemic Burkitt’s lymphoma, and posttransplant lymphoma (Maier et al., 2006). Moreover, increased expression of ABHD6 is found in U2OS (bone), Jurkat (leukocyte), PC-3 (prostate) and other tumor cell lines (Li et al., 2009). Abnormally high expression of ABHD6 is also observed in Ewing family tumors (EFTs) but not in other sarcomas, suggesting that it may be a new diagnostic target for these tumors (Max et al., 2009). Recently, Tang et al. found that ABHD6 plays a major role as an MAG lipase and oncogene in nonsmall-cell lung cancer (NSCLC) (Tang et al., 2020). ABHD6 correlates significantly with the tumor lymph node metastasis stage, which indicates a poor overall survival in NSCLC patients. Notably, ABHD6 silencing reduces the migration and invasion of NSCLC cells *in vitro* as well as metastasis and tumor growth *in vivo*. In contrast, ectopic overexpression of ABHD6 stimulates its pathogenic potential (Tang et al., 2020). ABHD6 is highly expressed in human and murine pancreatic ductal adenocarcinoma (PDAC) tissues and cells. PDAC is one of the most lethal cancers with a high metastasis rate (Gruner et al., 2016), and pharmacological and genetic inhibition of ABHD6 confirms reduced PADC cell proliferation *in vitro* and tumor metastasis *in vivo* (Gruner et al., 2016). Hepatocellular carcinoma (HCC) is one of the most common malignant tumors, and Yu et al. conducted a systematic transcriptome study on HCC, determining that the methylation level of zinc finger and SCAN domain-containing 18 (ZSCAN18) can be used as an indicator for HCC prognosis and that ABHD6 is a potential tumor suppressor (Yu et al., 2016). These results indicate expression of ABHD6 in malignant tumors. Overall, it is very important to confirm whether ABHD6 acts as a diagnostic marker and therapeutic target for malignant tumors.

## ABHD6 and Neurological Diseases

ABHD6 regulates eCB signaling by degrading the key lipid messenger 2-AG, which controls appetite, pain and learning and is associated with Alzheimer’s disease and Parkinson’s disease (Bleffert et al., 2019). Various studies have shown the therapeutic potential of targeting ABHD6 in the treatment of CNS diseases. Chronic pharmacological inhibition of MAGL is known to cause 2-AG overload, partial desensitization of CB_1_R, and loss of cannabinoid-mediated effects in specific brain regions, leading to adverse side effects such as low activity and hyperreflexia (Schlosburg et al., 2010). In contrast to MAGL targeting, genetic or pharmacological blockade of ABHD6 results in moderate accumulation of 2-AG without CB1-related side effects (Alhouayek et al., 2013; Deng and Li, 2020b). Therefore, inhibition of ABHD6 may help in preventing CB_1_R desensitization, making ABHD6 a promising new pharmacological target for the treatment of neurological diseases (Table 1).

**TABLE 1:** Overview of reported neurological disease treatments targeting ABHD6.

Disease	Therapeutic methods	Therapeutic effect
Traumatic Brain Injury (TBI)	ABHD6 Inhibitor (WWL-70)	improved motor coordination and working memory performance (mouse model)
Multiple Sclerosis (MS)	ABHD6 Inhibitor (WWL-70, KT-182)	reduced production of iNOS, COX-2, TNF-a and IL-1b, as well as phosphorylation of NF-kB (mouse model)
Epilepsy	ABHD6 Inhibitor (WWL-123)	significantly decreased seizure frequency (mouse model)
Psychiatric Disorders	Transcriptional Inhibition of ABHD6	terminating the stress response and inhibiting excitation after anxiety (mouse model)
Neuropathic Pain	ABHD6 Inhibitor (WWL-70)	significantly reduced thermal hyperalgesia and mechanical allodynia (mouse model)

### ABHD6 and TBI

ABHD6 inhibitors are beneficial to patients with traumatic brain injury (TBI). In TBI, secondary injury mediated by excitotoxicity, neuroinflammation, and oxidative stress is partially a result of an insufficient increase in 2-AG that fails to counteract these pathological processes (Schurman and Lichtman, 2017). WWL-70, an ABHD6 inhibitor, improves motor coordination and working memory performance in mice with TBI but does not affect spatial learning or memory impairment. Although WWL-70 has been used to explore the role of ABHD6 inhibition in TBI, genetic tools and more selective ABHD6 inhibitors need to be applied for verification (Tchantchou and Zhang, 2013).

### ABHD6 and Multiple Sclerosis

Multiple sclerosis (MS) is a chronic inflammatory disease that involves demyelination and axonal degeneration (Faissner et al., 2019). The histopathological manifestations of MS involve immune-dependent attack of oligodendrocytes and primary oligodendrocyte death (Jakel et al., 2019). The ECS plays a key role in the control of autoimmune demyelination. Cannabinoid drugs have therapeutic potential for MS patients (Goncalves and Dutra, 2019), but they also have limitations. Within this context, there is increasing evidence that hydrolysis of 2-AG, a major eCB, may tip the benefit-risk balance in favor of the use of 2-AG over the use of existing cannabinoid drugs for MS treatment (Manterola et al., 2018a). Based on the observation that WWL-70 plays a protective anti-inflammatory role in experimental autoimmune encephalomyelitis (EAE), blocking ABHD6 is considered to be a new strategy for the treatment of MS. Furthermore, Wen et al. found that inhibition of ABHD6 in an MS mouse model can improve the clinical symptoms of cerebral hemorrhage, indicating the therapeutic effect of targeting ABHD6 on MS (Wen et al., 2015). Nevertheless, recent data indicate that ABHD6 blockade exerts only modest therapeutic effects against autoimmune demyelination, which calls into question its utility as a novel therapeutic target in MS (Manterola et al., 2018b).

### ABHD6 and Epilepsy

Pharmacological inhibition of ABHD6 has an antiepileptic role in pentylenetetrazol-induced epilepsy and spontaneous epilepsy mouse models (Naydenov et al., 2014). Studies have shown that ABHD6 blockers regulate activity-dependent 2-AG production and subsequent CB_1_R activation, which is characteristic of some forms of epilepsy (Marrs et al., 2010). Inhibition of ABHD6 by WWL-123 significantly reduces the frequency of chemically and genetically induced seizures in mice (Naydenov et al., 2014), though this inhibitory effect that reduces seizure frequency is likely due to increased GABAA receptor activity and not CB_1_R or CB_2_R activation. This suggests that ABHD6 inhibitors inhibit excessive excitatory transmission in epileptic seizures through two mechanisms, providing a new entry point for clinical treatment. For example, ABHD6 elevates the level of endogenous ligand 2-AG, not that of the GABAA receptor, or it can allosterically increase GABAA receptor signal transduction, and not directly target receptor-binding sites. In either case, the mechanism reduces treatment tolerance.

### ABHD6 and Psychiatric Disorders

Over the past decade, much evidence has consistently strengthened the link between life stress and the prevalence of mood and anxiety disorders (Pizzagalli, 2014). Although acute environmental stress rarely causes long-term neurophysiological or behavioral changes (Rusconi and Battaglioli, 2018), chronic stress has a strong toxic effect on glutamatergic synapses (Yang et al., 2020). Physiological changes caused by chronic stress are considered to be the core characteristics of neuropsychiatric disorders (Zhang et al., 2019), and the ECS plays a key role in the homeostasis of acute stress. In particular, stress induces an increase in 2-AG synthesis (Morena et al., 2016). 2-AG stimulates presynaptic CB_1_R and inhibits glutamate release, terminating the stress response and inhibiting excitation after anxiety. Longaretti et al. revealed that ECS-mediated synaptic regulation is mediated by transcriptional inhibition of ABHD6 and MAGL in response to acute psychosocial stress in the mouse hippocampus (Longaretti et al., 2020a). This process is coordinated by the epigenetic corepressor lysine-specific demethylase 1A (LSD1, also named KDM1A), which directly interacts with the promoter regulatory regions of the ABHD6 and MAGL genes (Longaretti et al., 2020b).

### ABHD6 and Pain

Wen et al. have shown that WWL-70 can significantly reduce thermal hyperalgesia and mechanical allodynia induced by chronic constriction injury (CCI) (Wen et al., 2018). Notably, no cannabinoid receptor antagonist to date is able to reverse the anti-injury and anti-inflammatory effects of WWL-70, suggesting that a novel mechanism is involved in the antinociceptive effect of the 2-AG catabolic enzyme ABHD6 inhibitor WWL-70. Indeed, WWL-70 treatment does not alter phosphorylation levels of 2-AG, AA or phospholipase A2 (cPLA2) in injured sciatic nerves but significantly inhibits production of prostaglandin E2 (PGE2) and expression of COX-2 and prostaglandin E synthase 2 (PGES2) (Wen et al., 2018). Because AA production and cPLA2 phosphorylation are not affected by WWL-70, it has been speculated that this inhibitor may interfere with the eicosanoid signaling cascade downstream of AA production by inhibiting prostaglandin synthase and prostanoid E (EP) receptor-mediated signal transduction. Hence, compared with COX inhibitors, such as nonsteroidal anti-inflammatory drugs (NSAIDs), which cause significant gastrointestinal and cardiovascular side effects, the use of WWL-70 may be a better treatment option for neuropathic pain.

## Conclusion and Perspectives

In the past decade, research on ABHD6 has enabled us to initially understand its molecular mechanism and biological function in the CNS. Furthermore, by employing ABHD6 inhibitors, we have been able to explore the potential effect of ABHD6 in the treatment of CNS diseases.

The number of signaling lipids and interacting proteins regulated by ABHD6 suggests that it has multiple functions and is positioned as a key molecular hub for regulating multiple signaling systems. Previous studies have provided a solid foundation for the establishment of this enzyme as a bona fide member of the ECS; however, ABHD6 regulates additional signaling systems through different mechanisms independent of eCBs, including GABAA and AMPAR.

Recent studies have reported that targeting ABHD6 may have many therapeutic benefits in the treatment of CNS diseases. First, ABHD6 can control the availability of 2-AG and subsequent activation of CB_1_R, suggesting the therapeutic potential of targeting ABHD6 in epilepsy. Nevertheless, further solid experiments are still needed to thoroughly explore the exact mechanism before ABHD6 inhibitors are applied in the treatment of epilepsy. In addition, selective ABHD6 inhibitors have been found to have the potential to ameliorate TBI and other neurological and neurodegenerative diseases. Although ABHD6 inhibitors can improve the clinical symptoms of MS in animal models, only a moderate therapeutic effect in humans has been shown. Therefore, using ABHD6 as a drug target for MS remains controversial.

Concerning the physiological function of ABHD6 outside the CNS, ABHD6 inhibitors may have therapeutic effects on metabolic disorders. For example, the role of ABHD6 in lipid metabolism suggests that some peripheral diseases may be attenuated by targeting ABHD6. Additionally, ABHD6 inhibition may have potential in anticancer therapy; studies have shown that this enzyme is highly expressed in several tumors, though the exact role of ABHD6 in cancer remains unclear. In the future, it will be imperative to comprehensively and systematically study the role of ABHD6 in cancer.

Because ABHD6 is widely expressed, its physiological and pathophysiological roles in different tissues need to be thoroughly elucidated in cell, animal and clinical studies. Overall, ABHD6 expression and function in different cells of the CNS have not been fully elucidated, especially in astrocytes and microglia. Furthermore, although ABHD6 is highly expressed in the brain, experiments on its activity in brain homogenates have indicated that it is responsible for only a small portion of the 2-AG that is, hydrolyzed. What are the main functional lipids of ABHD6 metabolism? With high mortality and morbidity, ischemic stroke has become a major health challenge. Activation of the ECS can alleviate cerebral ischemia injury, for which the initiating factor is energy deficiency, and it needs to be determined whether energy deficiency after cerebral ischemia changes CPT1 regulation of ABHD6 enzyme activity. In summary, in-depth exploration of the ABHD6 mechanism of action in the CNS will provide not only a new theoretical basis for the occurrence and development of CNS diseases but also a new target for their treatment.
